# Morphology and Thermal Properties of Calcium Alginate/Reduced Graphene Oxide Composites

**DOI:** 10.3390/polym10090990

**Published:** 2018-09-05

**Authors:** Wanting Zhao, Yan Qi, Yue Wang, Yun Xue, Peng Xu, Zichao Li, Qun Li

**Affiliations:** 1 College of Chemistry and Chemical Engineering, Qingdao University, Qingdao 266071, China; 2016020406@qdu.edu.cn (W.Z.); qiyanqdu@126.com (Y.Q.); wangyue@qdu.edu.cn (Y.W.); xueyun@qdu.edu.cn (Y.X.); 2017020860@qdu.edu.cn (P.X.); 2 College of Life Sciences, Qingdao University, Qingdao 266071, China

**Keywords:** calcium alginate, reduced graphene oxide, composites, morphology, thermal stability

## Abstract

Calcium alginate (CaAlg) is a kind of biodegradable and eco-friendly functional material, and CaAlg/reduced graphene oxide (rGO) composites are expected to be applied as new textile, heat-generating, and flame-retardant materials. In this paper, the CaAlg/rGO composites were prepared by a sol-gel method and their morphological and thermal properties were studied. The results showed that the introduction of rGO can efficiently improve the thermal stability of CaAlg. Further study showed that rGO increased the carbon formation rate by 4.1%, indicating that the thermal stability was improved by the promotion of carbon formation. Moreover, the weight loss rate of the composites was faster at 180–200 °C than that of CaAlg, after which the rate was less comparatively, suggesting the better thermal stability of the composite. This maybe because the high heat transfer efficiency of rGO allowed the material to reach the temperature of the thermal decomposition of the glycan molecule chain within a short time, and then promoted carbon formation. The thermal cracking mechanism of the composites is proposed based on the experimental data.

## 1. Introduction

As a hydrophilic and soluble marine polysaccharide macromolecule [1,2], sodium alginate (NaAlg) can form a cross-linked network by interacting with multivalent cations [3] and improve their water and mechanical resistance, barrier properties, cohesion and rigidity [4,5,6]. Due to its biocompatibility, non-toxicity [7], low cost, and the ability to achieve functional gel through the addition of polyvalent cations, NaAlg has been developed for many applicable purposes [8,9,10,11,12].

Reduced graphene oxide (rGO) is a high-temperature resistant and environmentally friendly material with a melting point of over 4000 K [13]. It shows important application prospects in advanced materials science [14], new energy and biomedicine, and is considered to be a revolutionary material [15] for the future, making it a current research hotspot. Huang et al. prepared polyvinyl alcohol/rGO nanocomposites by a solution blending method, and the results showed that the composite materials burned to form a dense and uniform carbon layer, which can prevent the thermal mass transfer and exchange of the combustible gas between the matrix material and the outside [16]. Han et al. reported the preparation of the polystyrene/rGO nanocomposites by melt blending and demonstrated that rGO can markedly improve the thermal stability and flame retardant of the polystyrene matrix [17]. And it was demonstrated that the oxides of rGO can improve the mechanical and thermal properties of the matrix materials NaAlg [18]. Furthermore, the hydrogels and aerogels used as adsorbents for ciprofloxacin in wastewater were prepared by Yu et al. by introducing the oxides of rGO into NaAlg, and it was found that the weight loss of the composites was significantly lower, compared with that of pure NaAlg [19]. The results of the aforementioned studies suggest that rGO and its oxides can improve the thermal stability of the matrix material NaAlg.

As far as we know, there has been no report regarding the effect of rGO on the thermal stability of calcium alginate (CaAlg). rGO exhibits the characteristic of excellent thermal stability. While the CaAlg formed by cross-linking with Ca^2+^ based on NaAlg has been reported to be a kind of inherent flame-retardant material, the glycosidic bonds are still easily broken at high temperature, leading to the poor thermal stability. Thus, in this study, rGO was introduced to CaAlg, and the CaAlg/rGO nanocomposites were prepared by a sol-gel method in a green and simple way. The materials were characterized by Fourier transform infrared spectroscopy (FT-IR), X-ray diffraction (XRD), scanning electron microscope (SEM) and thermal gravimetric analysis (TGA), and the morphology and thermal stability were studied. Moreover, the thermal degradation mechanism of the nanocomposites was proposed based on the experimental data.

## 2. Experimental

### 2.1. Materials

Sodium alginate (biological grade) was purchased from Zhiyuan Chemical Reagent Co. Ltd. (Tianjin, China). Calcium chloride anhydrous (analytical grade) was purchased from Beichen Fangzheng Reagent Factory (Tianjin, China). Reduced graphene oxide (rGO) was provided by the Institute for Graphene Applied Technology Innovation of Qingdao University (Qingdao, China). All chemicals were used as received without any further purification. All solutions were prepared using deionized water.

### 2.2. Preparation of the CaAlg

**Scheme 1:**
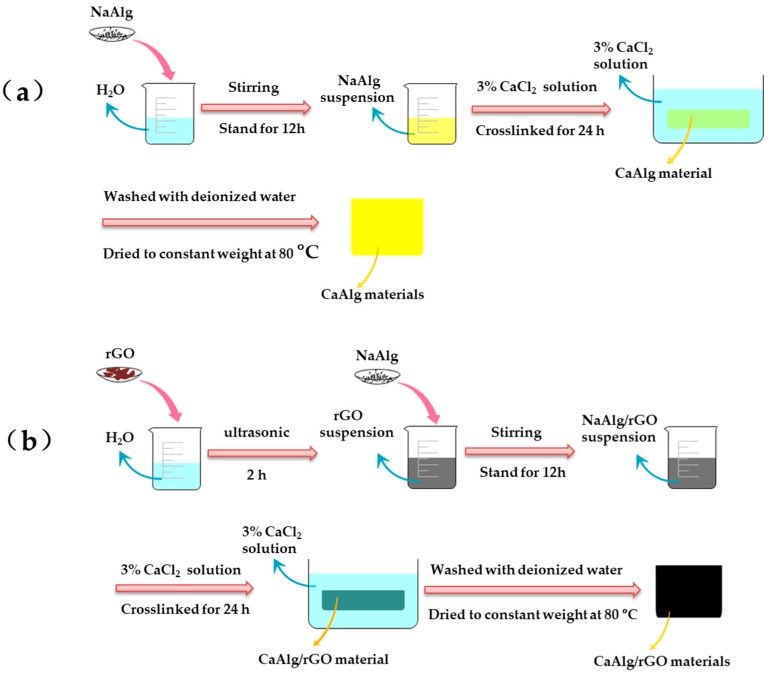
Schematic diagram of the preparation process of the (**a**) Calcium alginate (CaAlg) and (**b**) Calcium alginate/ Reduced graphene oxide (CaAlg/rGO) materials.

The preparation process of the CaAlg material is shown in Scheme 1a. NaAlg of 2.5 g was dissolved into 47.5 mL deionized water and stirred to form a homogeneous suspension, standing for 12 h to be de-foamed afterward. Then 300 mL of 3% CaCl_2_ solution was prepared. Subsequently, the NaAlg suspension was molded by a square mold, and poured into 3% CaCl_2_ solution and crosslinked for 24 h to be solidified. Finally, the solution was washed several times with deionized water and then placed into a drying oven (DHG-9070A, Yuhua Instrument Co., Ltd., Gongyi, China) at 80 °C to dry to a constant weight.

### 2.3. Preparation of the CaAlg/rGO Nanocomposites

The preparation process of the CaAlg/rGO material is shown in Scheme 1b. rGO of 50 mg was added into 47.5 mL deionized water with ultrasonic bath (KUOS, Kedao Supersonic Instrument Co., Ltd., Shanghai, China) for 2 h, and then was dispersed evenly in deionized water. The solution was slowly added with 2.5 g of NaAlg into a suspension and stirred to be dissolved to form a homogeneously mixed suspension, standing for 12 h to be de-foamed afterward. Subsequently, 300 mL of 3% CaCl_2_ solution was prepared, and the NaAlg/rGO suspension was molded by a square mold. And then it was poured into 3% CaCl_2_ solution and crosslinked for 24 h to be solidified. Finally, the solution was washed several times with deionized water and then placed into the drying oven at 80 °C to be dried to a constant weight.

### 2.4. Characterizations

#### 2.4.1. FT-IR

FT-IR (NICOLET iS50, Thermo Fisher Scientific Inc., Waltham, MA, USA) was recorded to confirm the chemical characteristics within the wavenumber range of 4000–500 cm^−1^.

#### 2.4.2. XRD

XRD was studied on an X-ray diffractometer (D/Max-RB, Rigaku Inc., Tokyo, Japan) using Cu-Kα radiation at room temperature. The samples were scanned up to 80° in 2θ in a continuous mode.

#### 2.4.3. SEM

SEM (JSM 6390LV, JEOL Inc., Tokyo, Japan) was employed to study the morphology and microstructure of the materials and their cracking residues. All the sample surfaces were sprayed with gold to prevent them from charging under the electron beam.

#### 2.4.4. TGA

TGA was analyzed on a thermogravimetric analyzer (TG 209, NETZSCH Geraetebau GmbH, Selb, Germany) with a heating rate of 10 °C/min. The prepared 5 mg of sample was determined with a flow rate of 50 mL/min from room temperature to 1000 °C under air and N_2_ atmosphere, respectively.

## 3. Results and Discussion

### 3.1. FT-IR Analysis

**Figure 1:**
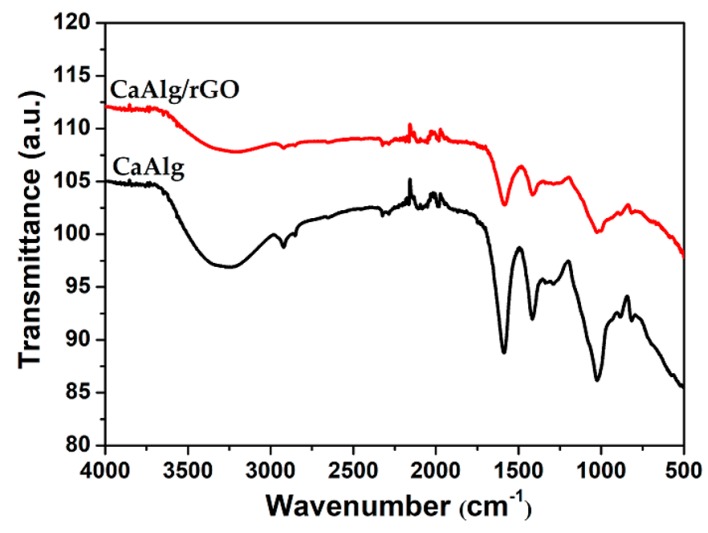
Fourier transform infrared spectroscopy (FT-IR) spectra of the CaAlg and CaAlg/rGO materials.

The FT-IR spectra of the CaAlg and CaAlg/rGO materials are shown in Figure 1. The main characteristic absorption peak positions and intensity of CaAlg and CaAlg/rGO were basically identical [20,21]. The broad and strong peaks at 3425 cm^−1^ can be ascribed to the stretching vibration absorption peak of –OH bond. The peaks at 2970 cm^−1^ corresponded to the stretching vibration absorption peak of –CH_2_ bond, and the peaks at 1629 cm^−1^ can be assigned to the asymmetric stretching vibration absorption peak of the –COO bond. The peaks at 1447 cm^−1^ were the in-plane bending vibration absorption peak of the –CH_2_ bond, and the peaks at 1045 cm^−1^ can be attributed to the stretching vibration absorption peak of the C–O–C bond [22]. Furthermore, with the addition of rGO, it can be noticed that the stretching vibration absorption peak of –OH bond at 3425 cm^−1^ was obviously broadened and the sharpness was decreased which can be due to the superposition of –OH on the surface of rGO and –OH of CaAlg molecular chain [23]. In addition, the asymmetric stretching vibration absorption peak of –COO bond at 1629 cm^−1^ was also broadened, caused by the superposition of –COO on the rGO surface and –COO on the CaAlg molecular chain. The oxygen-containing functional groups, such as –OH and –COO on the surface of rGO formed an interfacial hydrogen bond with the CaAlg molecular chain, which can induce close adhesion on the CaAlg and rGO surfaces. Moreover, each carbon atom in rGO was sp^2^ hybridized and contributed to the electrons in the remaining one p orbital to form the delocalized π bond. The π electrons can move freely, while the –OH of the CaAlg surface exhibited an electron-donating effect and can enhance the π-electron cloud density of the conjugated system, thereby inducing a close adhesion of rGO on the CaAlg surface.

### 3.2. XRD Analysis

**Figure 2:**
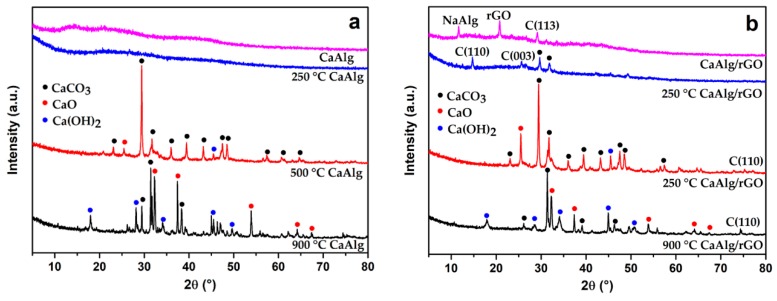
X-ray diffraction (XRD) patterns of the (**a**) CaAlg and (**b**) CaAlg/rGO nanocomposites.

**Figure 3:**
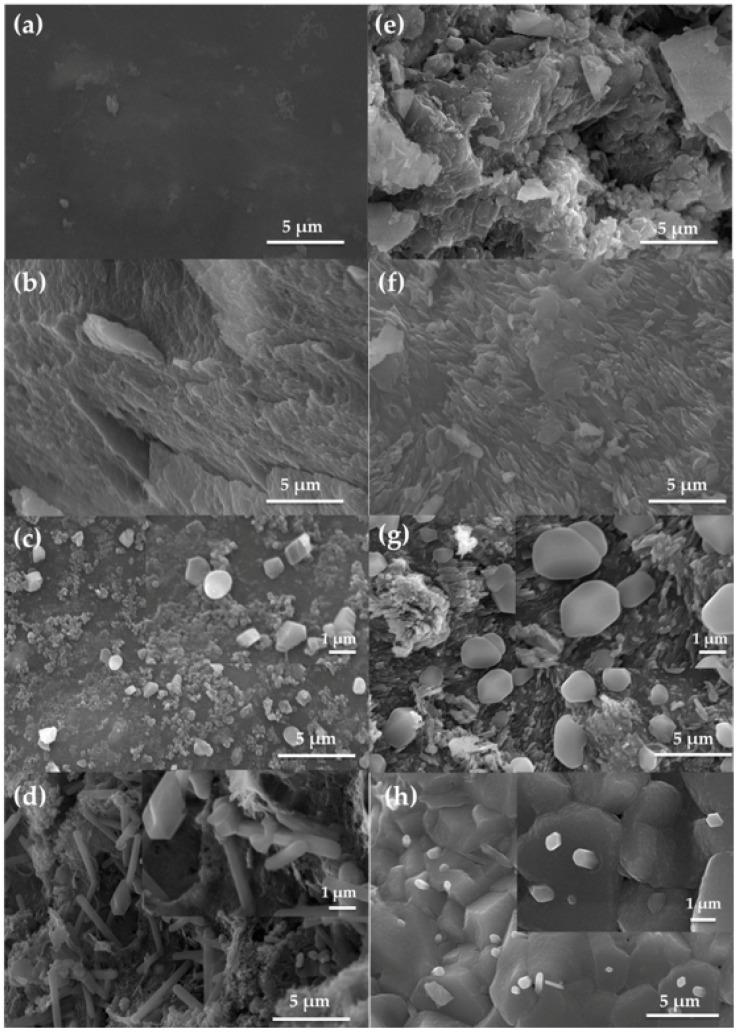
Scanning electron microscope (SEM) images of (**a**) CaAlg, (**b**) CaAlg at 250 °C, (**c**) CaAlg at 500 °C, (**d**) CaAlg at 900 °C; (**e**) CaAlg/rGO, (**f**) CaAlg/rGO at 250 °C, (**g**) CaAlg/rGO at 500 °C, and (**h**) CaAlg/rGO at 900 °C.

Figure 2 displays the XRD patterns of the CaAlg and CaAlg/rGO materials. As seen, two major diffraction peaks were observed in the CaAlg, namely a weak diffraction peak around 13.3° and a diffracted peak between 20° and 30°, while the diffraction peak of rGO was observed at 23.5° [24,25]. It can be noticed from Figure 2a that the CaAlg was a high molecular material with certain crystallinity, and its cleavage product at 250 °C was non-crystalline material, while the ones at 500 °C were CaCO_3_ and Ca(OH)_2_, and the ones at 900 °C were CaCO_3_, Ca(OH)_2_, and CaO, respectively. Notably, as seen in Figure 3b, the addition of rGO made the peak of CaAlg around 13° and 23° sharp and strong. Moreover, the characteristic peaks of residual carbon and CaCO_3_ in the cleavage product can be observed at 250 °C, and the peaks of CaCO_3_ and Ca(OH)_2_ at 500 °C, while the peaks of CaCO_3_, Ca(OH)_2_, and CaO were seen at 900 °C, respectively.

### 3.3. SEM Analysis

SEM images of the CaAlg and CaAlg/rGO materials are shown in Figure 3. As seen from Figure 3a,e, the surface of the CaAlg was relatively smooth, and it became rough with the addition of rGO, which may be in the reason that the slices of rGO were intercalated into the CaAlg [26,27]. As shown in Figure 3b,f, the surfaces of the two materials became rough and loose at 250 °C. However, the change of the CaAlg surface was not notable with the introduction of rGO, indicating its role in improving the thermal stability of the CaAlg. As seen from Figure 3c,g, many sphere-like crystals, as well as a small amount of irregular spherical crystals and square crystals were observed at 500 °C, while the diffraction peak of CaCO_3_, Ca(OH)_2_, and CaO can be seen from the XRD results. The results suggest that the CaAlg was decomposed into CaCO3 and Ca(OH)_2_ at 500 °C with the generation of little CaO. At 900 °C, lots of clustered spherical crystals, some square crystals and rod-like crystals intercalated on the top, as observed in Figure 3d. According to the diffraction peak in Figure 2a, it can be speculated that the clustered spherical crystals were CaO, and the square crystals were CaCO_3_, while the rod-like crystals were Ca(OH)_2_.

Furthermore, it can be observed from Figure 3h that the cracking products were relatively well-ordered square crystals, while the embedded spherical crystals and few rod-like crystals can be noticed, as the CaAlg was introduced with rGO and calcined at 900 °C. Combining the XRD patterns shown in Figure 2b, it can be inferred that the orderly arranged square crystals should be CaCO_3_, while the rod-like ones were Ca(OH)_2_ and spherical ones were CaO, suggesting that CaCO_3_ in the composites was not decomposed into CaO even at high temperature. Furthermore, it can be speculated that the carbon formation of the rGO-CaAlg system can cover the surface of the material, and block the thermal mass transfer and the exchange of the combustible gas between the matrix material and the outside. Thus, it may well play a protective role for the matrix material, and improve its thermal stability.

### 3.4. TGA Analysis

#### 3.4.1. Thermal Degradation Behaviors under N_2_ Atmosphere

**Figure 4:**
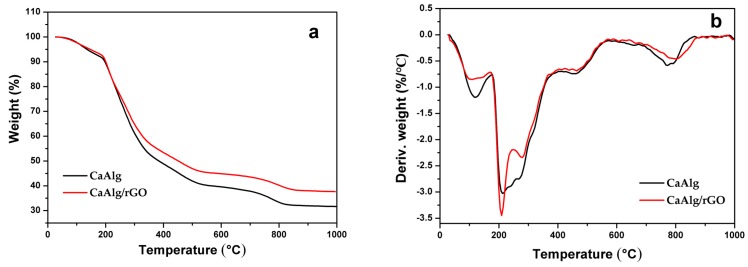
(**a**) Thermal gravimetric analysis (TGA) and (**b**) differential thermogravimetric analysis (DTG) of the CaAlg and CaAlg/rGO materials under N_2_ atmosphere.

The thermal stability of the prepared materials under an N_2_ environment is shown in Figure 4. As seen from Figure 4a, the curve of the CaAlg/rGO ran above the CaAlg since heating, which showed less thermal degradation than CaAlg did, indicating better thermal stability.

As shown in Figure 4b, the curve of the CaAlg/rGO was above the CaAlg at the beginning of heating, indicating that the thermal degradation rate of the CaAlg/rGO was lower than that of the CaAlg. At about 80 °C, both of the materials began to exhibit the first weight loss, while CaAlg/rGO showed a peak weight loss of approximately 3% at 100 °C. The weight loss peak of the CaAlg lagged behind the CaAlg/rGO slightly, and the peak weight loss around 110 °C was about 4%. Between 100 °C and 150 °C, the weight loss peak of the CaAlg was sharper than that of the CaAlg/rGO, suggesting its moisture content was higher than that of the CaAlg/rGO. Thus it can be inferred that the CaAlg/rGO absorbed less water than the CaAlg did, indicating that the intervention of rGO reduced the polarity and hygroscopicity of the CaAlg. At 150 °C, the two materials began to lose weight for the second time. The weight loss peak of the CaAlg/rGO was sharper than that of the CaAlg, suggesting a higher weight loss rate of the CaAlg/rGO, compared to that of the CaAlg. And at 250 °C, the CaAlg/rGO continued to be decomposed, with a peak weight loss of 30% at 280 °C, whereas the CaAlg was only 20% at 220 °C. This was because after adding rGO, the thermal conductivity of the CaAlg was improved, and the heat transfer rate was fast, while the decomposition rate of CaAlg in the material was accelerated. As a result, the weight loss rate of the CaAlg/rGO was higher than that of the CaAlg between 150 °C and 400 °C. However, the thermal weight loss of the CaAlg/rGO was still lower than that of the CaAlg, indicating that rGO can promote the carbon formation of the CaAlg.

At 400–600 °C, the weight loss rates of the rGO/CaAlg and CaAlg remained basically the same. Moreover, the weight losses of the two materials also occurred at 600 °C. The weight loss rate of the CaAlg, which was 65% at 780 °C, was higher than that of the CaAlg/rGO. The weight loss peak of the CaAlg/rGO lagged slightly, and a weight loss of 60% occurred at 800 °C. After the degradation reaction was finished, the curve remained unchanged. Thus, it can be speculated the degradation mechanisms of the CaAlg/rGO and CaAlg were different. Considering thermal stability, the weight loss of the CaAlg/rGO at 300–800 °C was apparently lower than that of the CaAlg, while the weight loss peak of which at 800 °C remarkably lagged behind that of the CaAlg. Therefore, with the addition of rGO, the thermal stability of the CaAlg was significantly improved.

#### 3.4.2. Thermal-Oxidative Degradation Behaviors under Air Atmosphere

**Figure 5:**
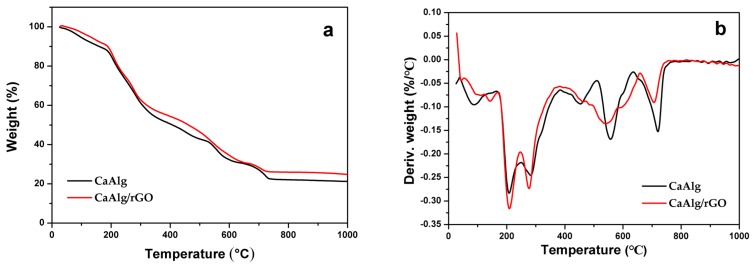
(**a**) TGA and (**b**) DTG of CaAlg and CaAlg/rGO materials under air atmosphere.

Figure 5 shows the TGA results under air atmosphere of the two materials. The thermal performances were similar to that under N_2_. It can be observed from Figure 5a that the curve of the CaAlg/rGO directly went above the CaAlg after heating, indicating that the CaAlg/rGO possessed better thermal stability than that of the CaAlg. However, as seen in Figure 5b, at 200 °C, the CaAlg/rGO exhibited a peak weight loss of 26%. Over 250 °C, the CaAlg/rGO continued to decompose with a 36% of weight loss at 280 °C. And the CaAlg showed a 27% weight loss peak at 200 °C, and it continued to be decomposed at 250 °C, and its peak weight loss at 290 °C was 40%. This was because after adding rGO, the thermal conductivity of the material was improved.

As a result, the weight loss rate of the CaAlg/rGO was higher than that of the CaAlg between 150 °C and 400 °C. However, the thermal weight loss of the CaAlg/rGO was still lower than that of the CaAlg, indicating that rGO promoted the carbon formation of the CaAlg. At 450 °C, the CaAlg showed a notable weight loss peak of 56%, while the peak of the CaAlg/rGO was about 50%. As the two materials continued to be burnt, remarkable weight loss peaks were observed at 540 °C. At 720 °C, the two materials exhibited weight loss again, and the weight loss rate of CaAlg was higher than that of the CaAlg/rGO. After the degradation reaction was over, the curve remained almost unchanged.

#### 3.4.3. Thermal Degradation Mechanisms

**Figure 6:**
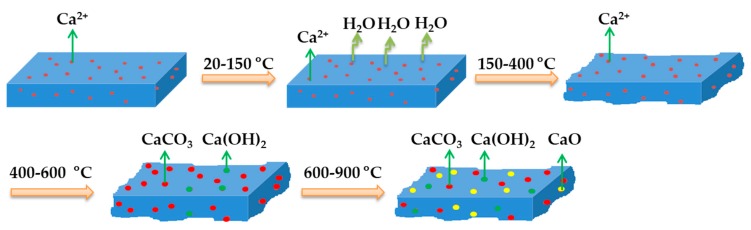
Schematic of the supposed thermal degradation mechanisms.

Based on the thermal cracking products of the two materials under N_2_ and air atmosphere, the thermal degradation mechanism was supposed and displayed in Figure 6, which is further discussed as follows:

1. The first stage of thermal degradation

First, the crystal water in the materials was lost at about 120 °C. At 120 °C, as shown in Table 1, the weight loss rate of the CaAlg was 3.9% while the CaAlg/rGO was 3.7%. It can be seen that the moisture content of the CaAlg was slightly higher, compared with that of the CaAlg/rGO. This shows that, when the moisture regain happened at room temperature, the CaAlg/rGO absorbed less water than the CaAlg did, suggesting that the intervention of rGO can reduce the polarity and hygroscopicity of the CaAlg. Both materials released gas water molecules in the early stage of combustion, which can dilute the air and, thus, reduce the burning rate.

2. The second stage of thermal degradation

As seen from TG curves (Figure 4 and Figure 5), the decomposition temperature of the CaAlg was about 250 °C. It can be observed from Table 2 that, at 250 °C, the weight loss rate of the CaAlg was 26.0% while the CaAlg/rGO was 24.0%, between which the difference was only 2.0%. As the amount of rGO added to the composites was 2.0%, thus rGO did not notably promote the carbon formation of the CaAlg at 250 °C. At 450 °C, the difference of the weight loss rate between the CaAlg and the CaAlg/rGO was 4.6%, indicating the carbonization rate of the composites increased by 2.6%. Up to 750 °C, the difference of the weight loss rose to 6.1%, and the carbonization rate reached 4.1%. The carbonization rate of the two materials did not change anymore when the temperature was higher than 850 °C. It can be inferred that introducing rGO to the CaAlg matrix can promote the rate carbonization, contributing to the enhanced thermal stability of the CaAlg/rGO, compared with that of the CaAlg. It shows that rGO can not only form carbon itself but also promote the carbon formation of the CaAlg. The formed carbon layer covered the surface of the materials, which blocked the thermal mass transfer and the exchange of the combustible gas between the materials and the outside. It can play a great protective role for the matrix material, and effectively improve its thermal stability. At this time, CO_2_ played the gas phase role, while the carbon formation of the rGO-CaAlg system, the endothermic decomposition of CaCO_3_ and the cover effect of CaO played the solid phase role together. It can be seen that the formation of the rGO-CaAlg system led to a change in the thermal degradation behavior of the CaAlg, as shown in Scheme 2.

**Scheme 3:**
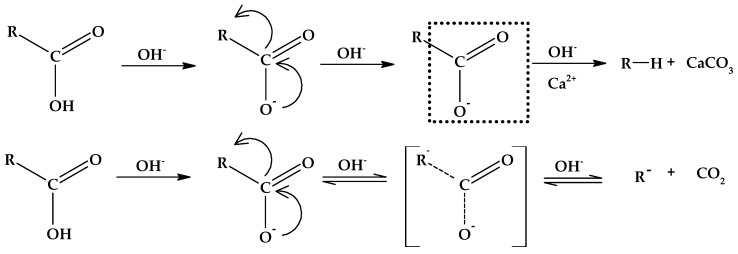
The schematic of decarboxylation mechanisms of the CaAlg and CaAlg/rGO materials.

The two materials can provide the ionization of carboxyl groups since they exhibited alkaline properties in the thermal cracking process, and, thus, the decarboxylation reaction of polysaccharide chains for the CaAlg was promoted while heating. Further description of the reaction is shown in Scheme 3, in which R represents the glycoside fragments generated from the decarboxylation of the macromolecular glycan.

## 4. Conclusions

The CaAlg/rGO nanocomposites were fabricated through introducing rGO into the CaAlg by a green and facile sol-gel method, and their morphological and thermal properties were studied. Moreover, the underlying mechanisms of thermal degradation were supposed based on the experimental data. It was found that after the addition of rGO, a synergistic system of rGO-CaAlg was formed, and the thermal stability of the system was remarkably improved. The introduction of rGO promoted the formation of carbon from the CaAlg, and the carbonization rate reached 4.1%. The formed carbon layer covered the surface of the substrate and blocked the thermal mass transfer and the exchange of the combustible gas between the matrix material and the outside. It played a very good protective role for the matrix material and improved its thermal stability. Furthermore, the prepared CaAlg/rGO composites are biodegradable and eco-friendly materials. Therefore, the materials can be promising when applied to the industry of new textile, heating, and flame retardant materials.

## Figures and Tables

**Scheme 2:** The schematic of the thermal degradation reactions of the CaAlg and CaAlg/rGO.

$$\begin{array}{l} \mathrm{CaAlg}\overset{\geq 250\;{}^{\circ}C}{\underset{\mathrm{decarboxylation}}{\to }}\mathrm{Alginate}\;\mathrm{decarboxylate}+{\mathrm{CO}}_{2}+{\mathrm{CaCO}}_{3}+{\mathrm{Ca}(\mathrm{OH})}_{2}\\ \mathrm{CaAlg}/\mathrm{rGO}\overset{\geq 250\;{}^{\circ}C}{\underset{\mathrm{decarboxylation}}{\to }}\mathrm{Alginate}\;\mathrm{decarboxylate}+{\mathrm{CO}}_{2}+{\mathrm{CaCO}}_{3}+{\mathrm{Ca}(\mathrm{OH})}_{2}+C\left(\mathrm{residual}\;\mathrm{carbon}\right)\\ {\mathrm{CaCO}}_{3}\overset{\geq 750\;{}^{\circ}C}{\underset{\mathrm{decomposition}}{\to }}\mathrm{Alginate}\;\mathrm{carbonization}+{\mathrm{CO}}_{2}+\mathrm{CaO}\\ {\mathrm{CaCO}}_{3}/{\mathrm{Ca}(\mathrm{OH})}_{2}/\mathrm{rGO}\overset{\geq 750\;{}^{\circ}C}{\underset{\mathrm{decomposition}}{\to }}\mathrm{Alginate}\;\mathrm{carbonization}+{\mathrm{CO}}_{2}+\mathrm{CaO}+C\left(\mathrm{residual}\;\mathrm{carbon}\right) \end{array}$$

**Table 1:** Comparison of weight losses of Calcium alginate (CaAlg) and Calcium alginate/Reduced graphene oxide (CaAlg/rGO) materials under N_2_ atmosphere before 200 °C.

Parameters	CaAlg	CaAlg/rGO	D-Value
Residues at 60 °C	99.6	99.3	0.03
Residues at 90 °C	98.3	98.0	0.03
Residues at 120 °C	96.1	96.3	0.02
Residues at 150 °C	93.8	94.6	0.08
Residues at 180 °C	92.0	92.9	0.09

**Table 2:** Comparison of the weight losses of the CaAlg and CaAlg/rGO materials under N_2_ atmosphere over 200 °C.

Paremeters	CaAlg	CaAlg/rGO	D-Value	Carbonization Rate
Residues at 250 °C	74.0	76.0	2.0	0
Residues at 350 °C	52.7	56.8	4.1	2.1%
Residues at 450 °C	45.2	49.8	4.6	2.6%
Residues at 550 °C	40.2	45.3	5.1	3.1%
Residues at 650 °C	38.8	44.3	5.5	3.5%
Residues at 750 °C	36.0	42.1	6.1	4.1%
Residues at 850 °C	32.2	38.3	6.1	4.1%
